# The Physician and the God of Love (Poem)

**Published:** 1875-01

**Authors:** 


### Our Exchanges.

[From the Clinic, November, 1874.

### *THE PHYSICIAN AND THE GOD OF LOVE.*

The doctor and the god of love
Are each on duty night and day.
Behold the resemblance.
One is famous in his age;
The other in his youthful days.
Behold the difference.

Of the two, each one is blind;
But each has still a curious mind.
Behold the resemblance.
One is grave, and black his dress;
The other bright in his nakedness.
Behold the difference.

To both mankind must have recourse,
Although they both are dangerous.
Behold the resemblance.
One gives pain our wounds to dress;
The other wounds with a caress.
Behold the difference.

Each of the two gives us new breath,
And each alike in life and death.
Behold the resemblance.
The doctor always has his cost;
But love once paid is ever lost.
Behold the difference.

Both look us in the eye to tell
If it goes ill, if it goes well.
Behold the resemblance.
The doctor seeks the pulse to test;
The heart is always Cupid's quest.
Behold the difference.

They come in haste, both god and man;
And each is somewhat charlatan.
Behold the resemblance.
One leaves us of disease bereft;
The other when there's nothing left.
Behold the difference.

